# Supplementary material for: Causal Relationship between Aging and Anorexia Nervosa: A White-Matter-Microstructure-Mediated Mendelian Randomization Analysis
Source: Biomedicines. 2024 Aug 16;12(8):1874. doi: 10.3390/biomedicines12081874 (PMC11351342; doi:10.3390/biomedicines12081874)
Supplement: Supplementary file 1 [file biomedicines-12-01874-s001.zip › Supplementary Figure/Figure S4/Singe SNP plot of MR analysis for TL to ubm-b-1725.pdf]

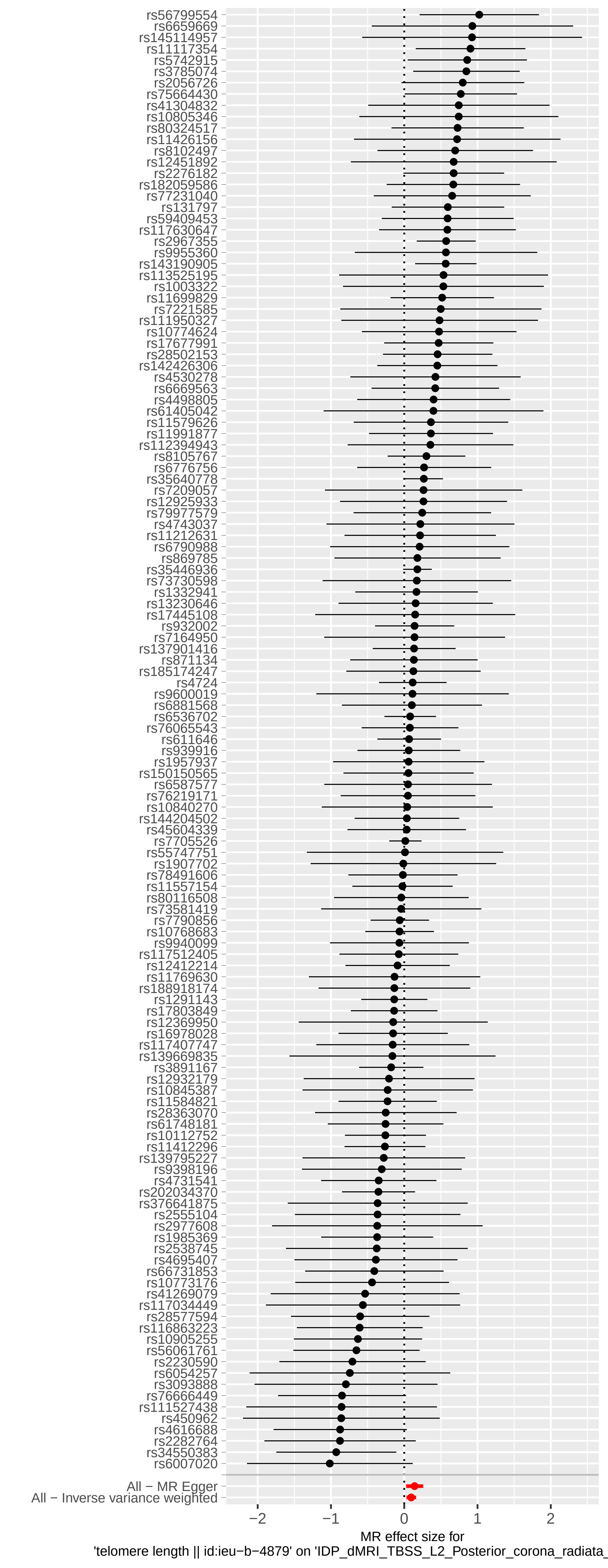
rs56799554
rs6659669
rs145114957
rs11117354
rs5742915
rs3785074
rs2056726
rs75664430
rs41304832
rs10805346
rs80324517
rs11426156
rs8102497
rs12451892
rs2276182
rs182059586
rs77231040
rs131797
rs59409453
rs117630647
rs2967355
rs9955360
rs143190905
rs113525195
rs1003322
rs11699829
rs7221585
rs111950327
rs10774624
rs17677991
rs28502153
rs142426306
rs4530278
rs6669563
rs4498805
rs61405042
rs11579626
rs11991877
rs112394943
rs8105767
rs6776756
rs35640778
rs7209057
rs12925933
rs79977579
rs4743037
rs11212631
rs6790988
rs869785
rs35446936
rs73730598
rs1332941
rs13230646
rs17445108
rs932002
rs7164950
rs137901416
rs871134
rs185174247
rs4724
rs9600019
rs6881568
rs6536702
rs76065543
rs611646
rs939916
rs1957937
rs150150565
rs6587577
rs76219171
rs10840270
rs144204502
rs45604339
rs7705526
rs55747751
rs1907702
rs78491606
rs11557154
rs80116508
rs73581419
rs7790856
rs10768683
rs9940099
rs117512405
rs12412214
rs11769630
rs188918174
rs1291143
rs17803849
rs12369950
rs16978028
rs117407747
rs139669835
rs3891167
rs12932179
rs10845387
rs11584821
rs28363070
rs61748181
rs10112752
rs11412296
rs139795227
rs9398196
rs4731541
rs202034370
rs376641875
rs2555104
rs2977608
rs1985369
rs2538745
rs4695407
rs66731853
rs10773176
rs41269079
rs117034449
rs28577594
rs116863223
rs10905255
rs56061761
rs2230590
rs6054257
rs3093888
rs76666449
rs111527438
rs450962
rs4616688
rs2282764
rs34550383
rs6007020
All − MR Egger
All − Inverse variance weighted
−2
−1
0
1
2
MR effect size for
'telomere length || id:ieu−b−4879' on 'IDP_dMRI_TBSS_L2_Posterior_corona_radiata_
